# Effective adaptations in Individual Placement and Support delivery: lessons from the drug and alcohol treatment context

**DOI:** 10.3389/ijph.2026.1609140

**Published:** 2026-08-03

**Authors:** Madeline Nightingale, Joanna Hofman, Kankan Zhang, Natalie Picken, Joanne Neale, Andrew Perkins

**Affiliations:** 1 RAND Europe, Cambridge, United Kingdom; 2 Ipsos MORI UK Ltd, London, United Kingdom; 3 Addictions Department, King’s College London, London, United Kingdom; 4 Figure 8 Consultancy Services Ltd, Dundee, United Kingdom

**Keywords:** adaptations, employment support, implementation science, IPS, substance use disorder

## Abstract

**Objectives:**

Guided by the Model for Adaptation Design and Impact (MADI), this paper explores how the Individual Placement and Support (IPS) model has been adapted for delivery in drug and alcohol treatment services in England.

**Methods:**

Interviews, focus groups and surveys with IPS practitioners, commissioners of drug and alcohol services, treatment service managers and experts.

**Results:**

Efforts to publicize the IPS service and sharing client ‘success stories’ with treatment staff are perceived to facilitate effective integration as a key implementation outcome. These adaptations also strengthen referrals to the IPS service, alongside changes to the referral process and schemes to incentivize referrals. Employer engagement is supported by careful framing of IPS and sharing client experiences and outcomes. Flexible and supportive communication with clients, adjusting the pace and taking breaks where needed, is perceived as crucial to achieving employment outcomes. Adaptations are shaped by the local and national context, including the scale of IPS delivery across England, which enables peer-to-peer networking and information sharing.

**Conclusion:**

Effective adaptations in IPS are those that strengthen integration, tackle stigma, tailor support, and enable learning networks.

## Introduction

Work can play an important role in recovery from drug and alcohol dependence, offering structure, financial stability, and reducing the risk of relapse [1, 2]. This is encouraging, as in 2023/24, only around one in three adults in the United Kingdom (UK) receiving drug or alcohol treatment were in regular employment (30%) [3], compared with 74.8% of the working-age population overall in 2024 [4]. There is also growing evidence that vocational interventions can improve treatment outcomes for this group [5, 6].

Individual Placement and Support (IPS) is an evidence-based, highly individualized form of employment support initially developed for people receiving treatment for severe mental illness [7–11]. The IPS model is built on eight core principles, including competitive employment as the goal, integration with treatment teams, zero exclusion (meaning all who want to work are eligible), attention to client preferences, benefits counselling, rapid job search, systematic job development (by building relationships with employers based on client preferences and local labor needs), and time-unlimited support. It is delivered by IPS Employment Specialists who are embedded within clinical teams to provide tailored and coordinated support [12].

IPS has been demonstrated to be effective for people receiving treatment for drug and alcohol dependence [13–17]. On the strength of this evidence, the 2021 UK Drugs Strategy [18] included a commitment to expand IPS delivery across all local authorities in England by March 2025. The structure and delivery of drug and alcohol treatment services in the UK differs from the mental health context. Specifically, locally commissioned treatment services are delivered by a network of third-sector, National Health Service (NHS) and local authority providers, often with multiple providers operating in the same area [19], creating potential challenges to co-location and integration. In addition, treatment clients often face distinct barriers, such as stigma, ongoing treatment requirements, co-occurring health problems, unstable housing, criminal convictions [20], often necessitating modifications in how Employment Specialists engage with and support clients, treatment staff, and employers.

Although IPS is a fidelity-based model [21], the drug and alcohol treatment contexts often differ from mental health services in which IPS is implemented and this in turn may affect the outcomes achieved [22]. Balancing fidelity to the IPS model with responsiveness to local conditions is critical to effective delivery. While some adaptations may improve engagement or outcomes, untested or inconsistent changes risk undermining program effectiveness. Without documenting these adaptations specific to treatment settings, it is unclear which changes improve, and which are detrimental to work outcomes, hindering the expansion of high-quality provision. Previous work on adaptations to IPS has focused primarily on mental health services [11, 23–25]. With IPS services in operation in drug and alcohol treatment across almost all local authorities in England, there is a timely need to capture and share learning on how the model is adapted in this treatment setting.

The aim of this article is to identify effective adaptations in IPS delivery in drug and alcohol treatment services in England and to explore how program delivery is tailored to and shaped by local contexts. The Model for Adaptation Design and Impact (MADI) is used to examine adaptations defined as “changes made to programs or interventions to align them with the context in which they are implemented” [22] and their perceived impact. By documenting adaptations that enhance integration with treatment teams, improve client engagement, and strengthen employer relationships, this study offers a practical framework for other services seeking to improve implementation and client outcomes.

## Methods

### Study design

This study used an iterative mixed-methods design drawing on: (1) semi-structured interviews, (2) focus groups and (3) an online survey. The participants and data collection tools for each method are described more fully below. Data were collected as part of two evaluations commissioned by the UK government: the process evaluation of the IPS for Alcohol and Drug Dependence (IPS-AD) trial [26] and the evaluation of the national expansion of IPS services [27].

### Theoretical framework

The MADI framework [22] is used to systematically identify, categorize, and interpret adaptations.

MADI provides a structured approach to describing adaptation characteristics (e.g., nature, timing, decision-making process, alignment with core functions) and assessing both their intended and unintended impacts on implementation and intervention outcomes. MADI was chosen because it not only documents which adaptations are made but also examines when and why they occur and how they may affect outcomes, offering a practical lens for balancing fidelity with flexibility in complex service settings.

### Participants

Data were gathered from stakeholders involved in the delivery of the trial and expansion of IPS in drug and alcohol treatment services in England (see Table 1). Purposeful sampling of interviewees and focus group participants aimed to ensure coverage of stakeholders with varied experiences of IPS implementation in both IPS and treatment teams [28]. Interviews were conducted with Employment Specialists and other IPS practitioners, commissioners of drug and alcohol treatment services and treatment staff. Focus groups were conducted with Employment Specialists, commissioners, treatment service managers and wider experts in IPS delivery. The sampling strategy for the interviews and focus groups was designed to ensure diversity across local authorities in terms of geographical location, length of time delivering IPS and type of drug and alcohol treatment service delivery (third sector, NHS, local authority). Employment Specialists from all IPS services that had been in operation for more than 6 months (some of whom may also have participated in the interviews) were invited (via email) by the evaluation team to participate in a survey based on a contact list provided by the relevant national authority, with two follow-up reminders sent to encourage participation.

**TABLE 1:** Overview of research participants (England, UK. 2018–2025).

Stakeholder group	Description		Number of participants	
		Semi- structured interviews	Focus groups	Survey
Employment Specialists	IPS team members. For simplicity, Senior Employment Specialists (team leaders) are grouped together with Employment Specialists	83	2 focus groups with 16 participants combined (all Senior Employment Specialists)	138
Other IPS practitioners	IPS lead at the provider or local level	7	n/a	n/a
Commissioners	Local authority staff responsible for commissioning drug and alcohol treatment services	30	1 focus group (joint with treatment staff) with 8 participants	n/a
Treatment staff	Managers of drug and alcohol treatment services and other members of staff	66	1 focus group (joint with commissioners) with 5 participants from treatment services (all managers of treatment services)	n/a
Experts	Experts in IPS and other forms of supported employment from within and beyond the drug and alcohol treatment context. This group included representatives from national government and academia	n/a	1 focus group with 7 participants from academia, national government and organisations supporting IPS delivery (IPS Grow, Social Finance)	n/a

### Data collection

Interviews were semi-structured, guided by standardized topic guides [29, 30] (a list of key questions can be found in the Supplementary Material and full topic guides are available from the corresponding authors upon request). This approach ensured methodological consistency across stakeholder groups while allowing flexibility to probe emerging issues. Interviews were conducted over the telephone or in person (interviewees could choose a face-to-face interview during site visits, where feasible and preferred) between July 2018 and October 2024. Focus groups were conducted online between November 2024 and February 2025. Focus groups examined strategies used to overcome challenges in IPS delivery, with interactive whiteboards used to stimulate discussion. An online survey was administered to Employment Specialists in October and November 2024, with 138 complete responses from 219 Employment Specialists who were invited to participate (a response rate of 63%). Survey questions were partly informed by emerging findings from the initial qualitative interviews, e.g., used to explore the prevalence of a view or experience and to understand which factors are perceived as more important or influential.

### Data analysis

Interview and focus group notes were verified against audio recordings and analyzed thematically. The coding framework was initially based on the evaluation questions, with additional themes added inductively [31, 32]. Further analysis was conducted using the MADI framework [22] to identify the main groups of adaptations and then to examine, in greater analytical detail, the related decision-making processes, implementation, moderators and mediators. Initial coding and analysis were conducted by a team of 10 researchers, with a structured template used to ensure consistency in qualitative analysis. Subsequent analysis using the MADI framework was undertaken by a smaller subgroup of three researchers, who revisited and synthesized data across datasets. Emerging interpretations and themes were discussed within the research team to support consistency in analysis. Formal assessment of data saturation was not undertaken; however, the research team observed substantial repetition in the main themes across stakeholder groups and data collection methods, which increased confidence in the study findings. All coders were researchers trained in qualitative analysis and were part of the evaluation teams (including being involved in conducting interviews and focus groups). Survey findings were analyzed descriptively, with both numbers and percentages reported given the relatively small number of responses. As questions were not always mandatory, totals vary across items. Qualitative and quantitative findings were interpreted together using the MADI framework, with survey findings used to contextualize and triangulate themes emerging from interviews and focus groups.

The research team had no involvement in IPS service delivery or local commissioning and no vested interest in specific implementation outcomes. Interpretation of findings was supported through discussion within a multidisciplinary research team and triangulation across multiple stakeholder perspectives and mixed methods data, rather than privileging a single analytic interpretation.

## Results

Stakeholder-reported adaptations to IPS are grouped into four categories: (1) strengthening integration with treatment services and improving referrals; (2) employer engagement and addressing stigma; (3) tailoring client support and (4) team management and knowledge sharing. Table 2 provides an overview of the identified adaptations and their characteristics guided by the MADI framework, including their perceived impact on implementation and intervention outcomes and potential moderators and mediators.

**TABLE 2:** Adaptations to Individual Placement and Support in drug and alcohol treatment (England, UK. 2018–2025).

Grouping	Domain 1					Domain 2	Domain 3
	What is modified (content; delivery; training & evaluation; implementation & scale up activities)	Nature of adaptation (adding, skipping, substituting elements; shortening, condensing pacing, repeating elements)	Who took part in adaptation decision-making	For whom/What is adaptation made	When did adaptation occur	Potential mediator/Moderator	Implementation/Intervention outcomes
(1) Strengthening integration with treatment services and improving referrals	Implementation: Preparation activities before IPS service established; Promotional and awareness-raising activities, including sharing success stories	Intensive site preparations, stakeholder engagement meetings Promotional activities include posters, leaflets, videos, newsletters and presentations about IPS in treatment services Using written case studies, videos, or informal updates to communicate positive client outcomes to treatment staff	Employment Specialists	Treatment managers; Treatment staff	Pre-implementation (planning stage) and implementation stage	Potential mediator(s): Improved awareness and understanding of IPS and greater ‘buy in’; Stigma reduction (in relation to perceptions of ‘work readiness’ from treatment staff) Potential moderator(s): Support from commissioner and treatment service manager	Implementation outcome(s): Improved integration with treatment services; increased referrals to IPS service; more suitable referrals
(1) Strengthening integration with treatment services and improving referrals	Implementation: Simplifying IPS referral process and/or widening referral pathways; incentivizing referrals	Simplifying referral process to reduce barriers (mode of referral; amount of information needed). Introducing incentives (e.g., reward or recognition for treatment staff making referrals, competition); Three-way appointments with treatment staff (‘warm hand over’); feedback about referrals; encouraging clients to self-refer (drop-in sessions, promotional materials, etc.)	Employment Specialists; Treatment managers	Treatment staff; IPS Service; Prospective IPS clients	Implementation stage	Potential moderator(s): Awareness of IPS and ‘buy in’ from treatment staff; visibility of IPS within services	Implementation outcome(s): Increased referrals to IPS service; more diverse client pool
(1) Strengthening integration with treatment services and improving referrals	Training: Training and on the job learning (e.g., shadowing) for treatment staff about IPS/employment	Formal training or on-the-job learning (e.g., shadowing Employment Specialists) to learn about employment and IPS; IPS being part of the induction for new treatment staff	Employment Specialists; Treatment managers	Treatment staff	Pre-implementation stage and implementation stage	Potential moderator(s): Previous exposure to IPS or employment support within the treatment provider	Implementation outcome(s): Improved integration with treatment services; increased referrals to IPS service; more suitable referrals
(2) Employer engagement and addressing stigma	Delivery: Careful framing of IPS to employers	Positioning (more general health/disability focus), language; branding (e.g., avoiding use of treatment-branded material or badges	Employment Specialists	Employers	Implementation stage	Potential mediator(s): Stigma (gradual introduction helps to tackle stigma) Potential moderator(s): Type of treatment provider(s) and pre-existing knowledge/awareness of provider(s) in employer population; client preferences regarding disclosure	Intervention outcome(s): More jobs available for and improved job outcomes (employment offer, starting work, sustained employment) for IPS clients
(2) Employer engagement and addressing stigma	Delivery: Sharing client experiences and client outcomes with employers	Facilitating contact between employers and IPS clients/those with lived experience; Sharing ‘success stories’ (e.g., case studies) with employers; Providing training for employers	Employment Specialists	Employers	Implementation stage	Potential mediator(s): Stigma (client stories and outcomes help to tackle stigma)	Intervention outcome(s): More jobs available for and improved job outcomes (employment offer, starting work, sustained employment) for IPS clients
(3) Tailoring client support	Delivery: Flexible support	Adjusting pace and support to client needs, especially where challenges are linked to treatment or wider life circumstances, while maintaining fidelity Flexibility in mode of contact, location of meetings (meeting in community) etc.	Employment Specialists	Clients	Implementation stage	Potential mediator(s): Relationship building and trust between employment specialist and client Potential moderator(s): Client specific factors/challenges faced	Intervention outcome(s): Improved engagement; Potentially higher job retention
(3) Tailoring client support	Delivery: Proactive strategies to maintain client engagement	Three-way appointments with treatment staff; meeting in the community; Tailored communication approach, e.g., use of COM-B framework; facilitating peer support/contact with other IPS clients	Employment Specialists; clinical psychologist; Treatment staff	Clients	Implementation stage	Potential moderator(s): Client anxiety, past rejection experiences; communication method; therapeutic relationship built up between employment specialist and client	Intervention outcome(s): Reduced non-attendance of appointments; sustained participation in IPS
(4) Team management and knowledge sharing	Training: Training provision and on the job learning (e.g., shadowing) for employment specialists related to drug and alcohol treatment and wider challenges faced by clients	Adding training on drug and alcohol awareness, challenges faced by clients on low doses of substitute prescribing, suicide and self-harm Prevention, promoting positive mental health, supporting people with criminal convictions into work, trauma-informed Practice, and domestic violence; on the job learning, including shadowing members of the treatment team	Employment Specialists; Treatment service managers	Employment Specialists	Implementation stage	Potential moderator(s): Professional background of employment specialist and prior knowledge and experience (including lived experience)	Implementation outcome(s): Expected to facilitate integration with treatment teams Intervention outcome(s): Expected to improve quality of client engagement
(4) Team management and knowledge sharing	Scale up activities: Sharing experience and good practice across IPS sites in drug and alcohol treatment and different IPS services (e.g., mental health, primary care)	Introducing telephone buddy system; Sharing resources; Peer mentoring networks; Sharing information about EE strategies and local employers	Employment Specialists; other IPS leaders (e.g., IPS Service lead for a provider)	Employment Specialists (including those embedded in other services)	Pre-implementation and implementation stage	Potential mediator(s): Problem-solving; improved performance of IPS teams Potential moderator(s): national IPS networks; prior experience and scale of operation for provider; IPS lead with oversight for multiple services	Intervention outcome(s): Expected to facilitate the spread of innovations and good practice; May improve consistency in delivery

### Strengthening integration with treatment services and improving referrals

Employment Specialists described efforts to raise awareness of the IPS service, including delivering presentations to treatment staff and using posters, leaflets and newsletters. This work sometimes began in the pre-implementation stage. Communication was sometimes tailored to different groups of treatment staff, for instance by using clinical language:

“We got good buy in from recovery practitioners but the clinical side we found very hard to break into (…) they were familiar with IPS in SMI [Severe Mental Illness] or in NHS settings and so we started with that, the language specifically we used was referring to IPS as a psychosocial intervention (…) [we thought they] might understand the benefits a bit better if it were in their language.” (Employment Specialist 86, focus group)

As well as strengthening integration with treatment services, these activities were designed to improve the quality and/or quantity of referrals to the IPS service. Employment Specialists reported that some treatment staff had reservations about job seeking and employment for certain clients, expressing concerns that this could destabilize or disrupt their recovery. Some treatment staff described how they would refer to IPS when a client had reached a certain stage in their treatment rather than being led solely by the client’s interest in work, as would be consistent with the zero-exclusion principle:

“If they’ve brought their alcohol use right down but they're at the end of that sort of phase and they're doing well in other aspects we’d be thinking about them working then.” (Treatment worker 60, interview)

Employment Specialists sharing client outcomes with treatment staff was perceived as highly effective in shifting mindsets about clients’ ‘readiness for work’, helping to increase referrals and strengthen compliance with the zero-exclusion principle of IPS. This could take the form of an informal discussion with treatment staff or an update in team meetings, but many IPS services had developed written or video case studies to share client stories and outcomes with treatment staff. All Employment Specialists surveyed regarded client success stories as an important factor in ensuring that treatment staff fully understand and engage with the IPS team and most (121/138) classed this as ‘very important’ (as opposed to moderately important or slightly important) (more detailed information about survey findings can be found in the Supplementary Material). Being able to have open and honest conversations with treatment staff to improve understanding and challenge misconceptions was similarly viewed as a very important factor by most of the Employment Specialists surveyed (123/138). Another common adaptation was training and on-the-job learning for treatment staff offered by IPS teams to improve understanding of IPS and the role of employment in recovery. Some IPS teams had developed microlearning courses to make the training as accessible as possible with a view to increasing engagement and improving uptake.

Other adaptations included changes to the referral process which were designed to make it quicker and easier, and in some cases, introducing schemes with token rewards for top referrers (in compliance with safeguarding and service ethics). Employment Specialists emphasized the importance of following up with referrers to provide feedback:

“Communication (…) is really important. Like broader communication to the whole service regularly, whether that be in like morning meetings or newsletters or any kind of like update. But then also on the back of each individual referral, whether or not you pick them up, whether or not you were unable to contact them, to always go back to that referrer and say this is how we got on with this person, this is the learning that we can take from it.” (Employment Specialist 86, focus group)

Employment Specialists encouraged clients to self-refer to the IPS service by hosting drop-in sessions at the local job center, attending group support sessions at the treatment service and distributing promotional materials designed for prospective clients. However, referrals from the treatment team, particularly in-person introductions, were perceived as particularly effective, allowing the IPS team to build on the trust and rapport already established between treatment worker and client:

“The referrals that are the most successful are the ones where we are physically introduced by the key worker: ‘*This is my colleague (…) She'd love to chat to you about your employment goals*’, and then I take them into a room from there and we talk about it, but they've already built that rapport and trust with that client and that won't hand over, just allows us to kind of pass over that trust immediately.” (Employment Specialist 89, focus group)

Stakeholders perceived that aspects of local context moderate the impact of these adaptations on integration and referrals. Not all Employment Specialists are employed directly by the treatment provider, are co-located with treatment services or have full access to their case management system. In some cases, stakeholders felt that it was easier to implement adaptations if these conditions were met, for instance co-location facilitated informal or *ad hoc* discussions with treatment staff and shared case management systems could make it easier to streamline processes for receiving referrals and providing feedback on referrals. Many Employment Specialists surveyed (79/138) agreed or strongly agreed with the statement ‘Integration is easier if IPS team members are employed by the same organization as the drug and alcohol treatment provider’. Interview data showed that being employed by the same organization was perceived to facilitate co-location, integration into treatment team meetings and access to case management systems, as well as fostering a sense of unity and team cohesion. However, some Employment Specialists surveyed (25/138) disagreed or strongly disagreed with this statement, indicating that successful integration is possible outside of this context. Support from institutional leadership, including local commissioners of drug and alcohol services and treatment service managers, was also perceived to be a key contextual factor.

### Employer engagement and addressing stigma

These adaptations focused on strengthening employer engagement and addressing stigma associated with hiring people in recovery, ultimately helping to expand the range and quality of job opportunities available for IPS clients. Employment Specialists reported that few of their clients wished to disclose their substance dependence to employers. As such, Employment Specialists were mindful of physical cues, for instance using plain rather than service-branded badges where the IPS provider might be immediately associated with the drug and alcohol treatment context. Branding was also used strategically to highlight a provider (or affiliated organization) expected to inspire trust in local employers, for instance an NHS affiliation. Employment Specialists adopted a careful framing of the IPS offer to employers, emphasizing its value in meeting genuine recruitment needs rather than portraying it solely as a support service for people with a history of substance dependence. The drug and alcohol dependence context was introduced—with client’s permission—gradually, with Employment Specialists often beginning with more general framing around health challenges:

“A lot of clients don’t want to disclose their substance misuse. (…) you can work intensively with employers to get them thinking about the client group that they make good employees, or you hide the fact that is a substance use service, and we are health service working with local people and we don’t really mention addiction, drug, alcohol. And we say that these people are currently unemployed with various health conditions and they want to go back to the job market. It is a tricky thing to negotiate.” (Employment Specialist 17, interview)

Once the substance dependence context was clear, Employment Specialists used various strategies to help tackle stigma and challenge misconceptions about this client group, including providing training for employers:

“We've provided training to a lot of local employers (…) we have done it in industries where we know that substance use is really commonplace like hospitality and finance and construction and laboring, we've given a lot of training to a lot of pubs in the area around how to talk to their employees about substance use in the workplace, how to challenge if you think that somebody has a problem with substance use in a supportive way, how to signpost people over to our services.” (Employment Specialist 86, focus group)

As with treatment staff, Employment Specialists shared compelling success stories with employers to challenge preconceptions. These narratives—delivered in written, video, or informal formats—humanized candidates and showcased the mutual benefits of IPS placements. Some Employment Specialists facilitated face-to-face contact between employers and IPS clients or those with lived experience:

“What is hugely valuable sometimes is if we can take a service user with us and talk to employers because you can hear a pin drop in the room, you know, when you’re actually listening to somebody with their own lived experience talking about it.” (Employment Specialist 64, interview)

Contextual mediators include strong local employer networks, which facilitate initial contact, and support from treatment staff. Lack of client’s disclosure of their substance use is a key moderator. Without disclosure from the client, Employment Specialists can only discuss employment in general terms with employers without linking this to an individual candidate or employee. Employment Specialists are also constrained in the in-work support they can offer without disclosure, being more limited in support they can offer to employers, including line managers, as well as clients.

### Tailoring client support

IPS is designed to be a personalized service, with Employment Specialists aiming to make a good match with the client’s interests, needs and preferences [12]. Employment Specialists emphasized the importance of delivering IPS in a flexible and individualised way, adjusting pace and support to client needs:

“Ensuring that what we do is in line with the client’s aims, rather than funneling them into whatever work is available, that builds rapport and trust and ensures they know we’re on their side. Understanding and rolling with the ebb and flow of clients’ motivation. The nature of the client group is that motivation can come and go in response to things happening in their lives. They may disappear for a while.” (Employment Specialist 43, interview)

Employment Specialists reported that it was relatively common for clients to disengage from the IPS service (e.g., not attending appointments or responding to messages), although many went on to re-engage. Employment Specialists described a range of adaptations designed to respond to weak or intermittent client engagement, including meeting the client out in the community rather than in the treatment center, having joint meetings with treatment staff and facilitating contact with local peer support services. Some Employment Specialists reported using tools such as the Capability, Opportunity, Motivation–Behavior (COM-B) framework [33] to take an evidence-informed approach to communication. In one case, an IPS team worked with a clinical psychologist to co-develop strategies to address non-attendance [26]. Evidence-informed changes to client communication included avoiding harsh, accusatory or punitive language, mentioning the names of individuals including treatment staff who had developed a good relationship with the client, and sharing statistics about the outcomes achieved by the service.

### Team management and knowledge sharing

Many IPS team leaders and commissioners of drug and alcohol services attributed positive outcomes achieved by their IPS service to the skills and qualities of team members. Some felt it was advantageous to recruit Employment Specialists with a background in drug and alcohol services, since this was perceived to help facilitate integration with treatment services. The value of having Employment Specialists with lived experience of substance dependence was also highlighted as a factor that could support effective relationship building with clients and help to tackle stigma. Other team leaders and commissioners felt it was more important to have team members with a background in employment support or IPS, or a mix of professional backgrounds within the team.

A common adaptation was training or upskilling related to drug and alcohol use and treatment for Employment Specialists, particularly those without a relevant background. Most Employment Specialists surveyed (110/135) reported receiving training in substance use or treatment provided by treatment services. All found it helpful, and over half (74/110) described this as ‘very helpful’. Training covered a range of topics including drug and alcohol awareness, treatment journeys and challenges faced by clients on low doses or substitute prescribing, as well as wider or interrelated issues, such as mental health, suicide prevention, domestic violence and supporting people with criminal convictions into work. Upskilling also took the form of more informal on-the-job learning, such as shadowing members of the treatment team.

Another adaptation widely perceived to support effective delivery was peer-to-peer learning and information sharing between IPS teams. Employment Specialists reported learning a great deal from other IPS teams, particularly those who had been in operation for longer:

“I spent a lot of time with [the team leader from a more established IPS service] learning about [their] role specifically and then we also spent time with the Employment Specialists learning about their role and also sharing documents, so they had vocational profiles, they had examples of good case (…) studies, employer engagement, so we were able to adopt a lot of their ways of working and a lot of their documents and fashion them then to our own systems and our own approach.” (Employment Specialist 53, interview)

As illustrated by the quotation above, Employment Specialists shared practical resources such as templates and documents as well as information and advice about effective strategies for employer engagement and client support. Some Employment Specialists described sharing information about local employers with other IPS services operating in the area (for instance, in mental health services), avoiding duplication of effort and preventing a situation where employers were overwhelmed with similar approaches:

“We've got a database of (…) employers (…) we have shared that with other IPS, local services, they've shared their database, and it's reduced the competition and it's all for the obviously benefit of the client. So that's just relieved a bit of tension and when we go out to see employers who have already been seen by other IPS employment services (…) it can make it to be easier rather than the employer thinking: *‘I've already spoke to one employer service. Do I need to speak to another one?’*” (Employment Specialist 87, interview)

Peer-to-peer networking is facilitated at the national level by the UK government and IPS Grow [34], as well as in some cases by the treatment provider. Some treatment and IPS providers operate across multiple local authorities and facilitate cross-team training and learning. In other local authorities, local governance structures were perceived to support effective co-working across different IPS teams (e.g., IPS teams in secondary SMI and primary care as well as drug and alcohol treatment). At both the provider and local authority level, there were examples of peer-to-peer networking facilitated by an IPS lead who had oversight over multiple IPS teams.

“Although we’re a little band of three in IPS AD, we’ve got that support of fifty other people and fifty other Employment Specialists [in the local authority] (…) We have fortnightly peer meetings. We have monthly business meetings within IPS (…) we promote very much learning from within and peer-to-peer support within our service (…) everybody within the IPS team supports everybody else.” (Employment Specialist 54, interview)

“They [SMI IPS team] just explained things because they had experience, so there’s nothing like lived experience of doing it and because they were so open to doing that they were lovely, so they just said how they'd gone about it, what they did, when we asked about the theory they told us how they did it in practice, they met with the Employment Specialists together, supported them a bit, they shared some of what they did, they were happy to share who they worked with as employers” (Commissioner 26, interview)

## Discussion

Building on earlier work that explores adaptations in IPS delivery [11, 23–25], this paper examines IPS adaptations in a new delivery context, that of drug and alcohol treatment services in England. Adaptations are described and analyzed according to the MADI framework [22], including their perceived impact on implementation and intervention outcomes and potential mediators and moderators.

The analysis draws on in-depth qualitative and quantitative data from a large, multi-site dataset, integrating multiple stakeholder perspectives. However, findings are based on stakeholder perceptions of successful adaptations rather than analysis that could demonstrate causal relationships. A risk of response bias must be acknowledged, since it is possible that stakeholders with certain views or experiences were more likely to participate in the research. There is also a risk that the mode of data collection (telephone vs. in person interviews) could have influenced participation patterns and responses [35]. Although efforts were made to engage with stakeholders involved in IPS delivery in diverse settings across the drug and alcohol treatment context, it is possible that a larger-scale study would have generated new or different insights. The intended delivery model for IPS in drug and alcohol treatment was consistent throughout the fieldwork period (2018–2025), but it is possible that adaptations and how these were perceived by stakeholders might have changed over this period, something it was not possible to explore as part of this analysis. Finally, although templates were used to promote consistency in qualitative coding and single-coder assignment supported consistency across each transcript, no independent double-coding or formal assessment of intercoder agreement was undertaken. As a result, the opportunity to identify and resolve potential coding errors through independent cross-checking was limited.

The main conclusion of this analysis is that IPS adaptations perceived to be most effective are those that strengthen integration, tackle stigma, tailor support, and enable learning networks. While IPS for individuals with SMI faces similar issues, how they are addressed in the drug and alcohol context is different because of the population served and the institutional context. Many of the identified adaptations align with the foundational principles of IPS, such as taking an integrated, collaborative and individualized approach, supporting the idea that adaptations are more likely to have a positive impact if they are aligned with the core principles of an intervention [22, 36]. The adaptations described in this paper do not compromise the basic principles of IPS [11] and are not perceived by stakeholders to undermine fidelity; in some cases, they are seen to support it. For instance, awareness raising activities and the provision of information and training for treatment staff are perceived to strengthen compliance with the zero-exclusion principle.

Adaptations are designed to improve the fit between intervention and context [22] and while the adaptations described in this paper respond to the specific context of drug and alcohol treatment in England, the findings have relevance for IPS practitioners in other contexts. Stigma is likely a common challenge for IPS clients in drug and alcohol treatment beyond the UK (with trials in the US [37], Norway [38], and Sweden [39]), as well as other groups supported by IPS, including people with common mental health conditions, autism, homelessness or criminal justice experience [11]. Some of the adaptations designed to address stigma—for instance, sharing client ‘success stories’ with treatment staff and employers—are likely to be low cost to implement and are perceived to be highly effective. Factors moderating the impact of adaptations such as whether Employment Specialists are employed directly by the treatment provider, co-location, access to case management systems and support from local authorities may also have relevance for IPS practitioners in other contexts, particularly where there is a complex commissioning structure.

The existence of learning communities has been identified as a factor facilitating the expansion of IPS in the US and UK contexts [26, 40, 41]. In this study, learning communities emerged as a key factor facilitating the sharing of resources and good practice, enabled by the scale of IPS delivery in England both within and beyond the drug and alcohol treatment context. Stakeholders involved in scaling up IPS and other interventions designed to support people with psychiatric disabilities into employment might consider how to encourage and enable peer-to-peer networking and mutual learning.
