# Supplementary material for: Effective adaptations in Individual Placement and Support delivery: lessons from the drug and alcohol treatment context
Source: Int J Public Health. 2026 Aug 3;71:1609140. doi: 10.3389/ijph.2026.1609140 (PMC13478017; doi:10.3389/ijph.2026.1609140)
Supplement: Supplementary file 1 [file DataSheet1.pdf]

Supplementary information

Example interview questions:

- What has worked well in terms of achieving seamless integration and what has worked less well?
- What has enabled integration (and physical co-location) in your area? What has made it more difficult?
- What has helped your team to deliver IPS with fidelity? What has made this more difficult?
- What strategies do you find most effective for working with IPS clients?
- To what extent are you able to offer support for clients that is integrated and coordinated with treatment services? What has enabled you to do this?
- What strategies do you find most effective for employer engagement and why?

Example focus group questions:

- When you were setting up your IPS service, what were some of the challenges you experienced and what kind of strategies did you use to address these?
- What are some of the challenges you have experienced in relation to integration and referrals and how have you addressed these?
- What strategies do you find most effective for employer engagement and why?
- What strategies do you find most effective for working with IPS clients?

Survey questions and responses:

In your view, how important are the following factors for ensuring that treatment staff fully understand and engage with your IPS team?

	Very important	***Moderately important***	***Slightly important***	***Not important***	***Not applicable/does not happen in my IPS service***	***Don't know***
IPS team members being employed by the same organisation as the treatment provider	64	19	16	34	3	2
The treatment provider(s) having prior knowledge and experience of IPS	58	45	22	11	2	0
Access to information, guidance and training provided by OHID, IPS Grow and other organisations	83	34	14	3	3	1
Networking and sharing information and advice with other IPS Employment Specialists	95	31	9	1	2	0
Being able to be physically present when needed in treatment services	106	24	5	1	2	0
Raising awareness of IPS in treatment services	114	17	3	0	4	0
Recruiting and retaining IPS team members with the right attributes, knowledge and skills for the job	121	11	2	0	4	0
Having open and honest conversations with members of the treatment team to improve understanding and challenge misconceptions	123	10	2	0	3	0
Sharing client success stories with the treatment team and employers	121	12	2	0	3	0

Note: based on n=138 Employment Specialists

To what extent do you disagree or agree with the following statements about integration and working with treatment services in your area?

	Strongly agree	***Agree***	***Neither agree nor disagree***	***Disagree***	***Strongly disagree***	***Don't know***
Integration is easier if IPS team members are employed by the same organisation as the drug and alcohol provider	49	30	25	13	12	9

Note: based on n=138 Employment Specialists

What training or support have you accessed in relation to your role in delivering IPS in drug and alcohol treatment and how helpful did you find this?

	Very helpful	***Moderately helpful***	***Slightly helpful***	***Not helpful***	***Not applicable/ did not access***	***Don't know***
Training in substance use/treatment arranged by treatment services	74	29	7	0	24	1

Note: based on n=135 Employment Specialists
